# Synthesis and Structural Activity Relationship Study of Antitubercular Carboxamides

**DOI:** 10.1155/2014/614808

**Published:** 2014-12-30

**Authors:** D. I. Ugwu, B. E. Ezema, F. U. Eze, D. I. Ugwuja

**Affiliations:** ^1^Department of Pure and Industrial Chemistry, University of Nigeria, Nsukka 410002, Nigeria; ^2^Department of Chemical Sciences, Federal University, Wukari, Nigeria

## Abstract

The unusual structure and chemical composition of the mycobacterial cell wall, the tedious duration of therapy, and resistance developed by the microorganism have made the recurrence of the disease multidrug resistance and extensive or extreme drug resistance. The prevalence of tuberculosis in synergy with HIV/AIDS epidemic augments the risk of developing the disease by 100-fold. The need to synthesize new drugs that will shorten the total duration of effective treatment and/or significantly reduce the dosage taken under DOTS supervision, improve on the treatment of multidrug-resistant tuberculosis which defies the treatment with isoniazid and rifampicin, and provide effective treatment for latent TB infections which is essential for eliminating tuberculosis prompted this review. In this review, we considered the synthesis and structure activity relationship study of carboxamide derivatives with antitubercular potential.

## 1. Introduction

Tuberculosis is an infectious disease caused by* Mycobacterium tuberculosis*, which most commonly affects the lungs. The* M. tuberculosis* complex is a set of evolutionary closely related slow growing mycobacterial species, all containing the mobile insertion sequence IS6610 in their genome and causing TB disease in humans and other mammals. It is transmitted from person to person via droplets from the throat and lungs of people with the active respiratory disease. In healthy people, infections with* Mycobacterium tuberculosis* often cause no symptoms since the person's immune system acts to “wall off” the bacteria. The symptoms of active TB of the lung are coughing, sometimes with sputum or blood, chest pains, weakness, weight loss, fever, and night sweats [1]. TB is a worldwide pandemic [2] and still remains one of the foremost among infectious diseases in the world causing the maximum number of deaths due to the spread of single microorganisms [3]. Of the new TB cases reported, 95% occur in developing countriesevery year. Currently, among the infected individuals, approximately eight million develop active TB, and almost two million die from the diseases [4]. The World Health Organization has expressed concern over the emergence of virulent drug-resistant strains of TB and is calling for measures to be strengthened and implemented to prevent the global spread of these deadly TB strains. The unusual structure and chemical composition of the mycobacterium cell wall and effective TB treatment is difficult, which makes many antibiotics ineffective and hinders the entry of drugs. Multidrug resistance tuberculosis (MDR-TB), defined as resistance to at least isoniazid and rifampicin [5], is a serious threat to tuberculosis control and prevention. Isoniazid blocks the biosynthesis of mycolic acids, the essential components of mycobacterial cell wall, and is believed to be oxidized by catalysed peroxidase (*Kat G*) to the active form [6]. Mutations in the* Kat G* and the* Inh A* genes are associated with 70–80% of INH-resistant* M. tuberculosis* isolate [7]. Resistance to rifampicin has been associated with mutations in the 81 bp core region of the* rpoB* gene encoding the *β*-subunit of RNA polymerase [8, 9] in over 90% cases. The development of resistance by* M. tuberculosis* to the commonly used antitubercular drugs necessitates a longer duration of therapy. The emergence of multidrug resistance has forced the development of new structural classes of antitubercular agents, with several of them showing promising activity against* M. tuberculosis *[10]. The tedious duration of therapy and resistance developed by the microorganism has made the recurrence of the disease especially as MDR-TB and XDR-TB [11]. A global challenge in tuberculosis chemotherapy XDR-TB is extensive or extreme drug resistance is MDR-TB that is also resistant to three or more of the six classes of second line drugs. The increase in TB incidence during recent years is largely due to the prevalence of TB in synergy with human immunodeficiency virus (HIV/AIDS) epidemic, which augments the risk of developing the disease by 100-fold, where 31% of new TB cases were attributed to HIV coinfection and emergence of MDR-TB and XDR-TB strains. The treatment of MDR-TB and XDR-TB has become a major concern worldwide. The occurrence of TB is linked to dense population, poor nutrition, and poor sanitation. Observed treatment short-course (DOTS) strategy constitutes the cornerstone of the current protocol for the control of TB [12–19]. Currently, the recommended standard chemotherapeutic regimen for TB treatment is prescribed under DOTS. The chemotherapeutic regimen consists of an initial 2-month phase of treatment with isoniazid (INH), rifampicin (RIF), pyrazinamide (PYR), and ethambutol (ETH) followed by a continuation phase of treatment lasting for 4 months with isoniazid and rifampicin. Poor patience compliance can promote the emergence of drug resistance, and this is particularly true in TB chemotherapy [20]. In the last forty years, only a few drugs have been approved by the Food and Drug Administration (FDA) to treat TB, reflecting the inherent difficulties in discovery and clinical testing of new agents and lack of pharmaceutical industry research in the area. There is unequivocal need for new drugs that should show improvement over the existing regimens in the following areas: (a) shortening the total duration of effective treatment and/or significantly reducing the total number of doses needed to be taken under DOTS supervision; (b) improving the treatment of MDR-TB, which cannot be treated with INH and RIF; and/or (c) providing more effective treatment of latent/dormant TB infection, which is essential for eliminating tuberculosis [21]. Recently, bedaquiline formerly known as TMC 207 was approved by United State Food and Drug Administration for the treatment of adult with pulmonary multidrug resistance tuberculosis when an effective treatment regimen cannot otherwise be provided [22]. It has no cross resistance to the available tuberculosis agents. Bedaquiline is marketed as SIRTURO and chemically known as (1R,2S)-1-(6-bromo-2-methoxy-3-quinolinyl)-4-(dimethylamino)-2-(1-naphthalenyl)-1-phenyl-2-butanol. Though this drug has good antitubercular activity, it needs 24 weeks of treatment thereby encouraging the development of resistance specie arising from noncompliance to prescription and it has also been shown to cause adverse effects like hemoptysis and anorexia [23]. Delamanid marketed as Deltyba is also indicated for use as part of an appropriate combination regimen for pulmonary MDR-TB in adult patients when an effective treatment regimen cannot otherwise be composed for reasons of resistance or tolerability. It must be administered as directly observed therapy (DOT) because of its adverse effect and it lasts for 24 weeks. There has been no established safe dosage for patients with renal or hepatic impairment and children or adolescents [24].

In an effort to discover new and effective chemotherapeutic agent for the treatment of TB, the antimycobacterial activities of various phthalazin-4-yl acetamides [25], thiazolylthiosemicarbazones [26], chromeno[3,2-c]pyridine-3-yl derivatives [27], [1,4]-thiazines [28], thieno-[2,3-b]thiophene [29], spirocyclohexanones derivatives [30], thieno[3,2-b]indoles [31], furan-2-yl derivatives [32], thiadiazoles derivatives [33], imidazole derivatives [34, 35], acyclic deoxy monosaccharide derivatives [36], benzoic acid hydrazine class [37], calanolide A, a naturally occurring coumarin derivatives [38, 39], purine derivatives [40, 41], pyrrole derivatives [42, 43], benzoxazine derivatives [44], diterpenoids derived from plants [45, 46], and quinoline and quinoxaline derivatives [47] have been reported.

## 2. Synthesis of Pyrazine Derived Carboxamides

Pyrazine carboxamide is an important component in the intensive phase of short-course treatment of TB owing to its sterilizing effect, ability to act in acidic environments, and excellent synergy with rifampicin. Martin et al. [48] synthesized binuclear analogues with the –CONH– bridge connecting the pyrazine and benzene rings with antimycobacterial activity. They proposed the formation of centrosymmetric dimer pairs with the peptidic carboxamido group of some peptides needed for binding to the receptor site possibly by hydrogen bond formation.

**Scheme 1:**
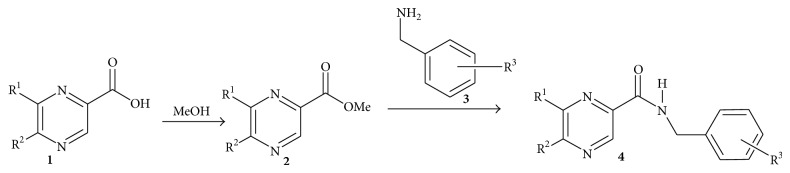
Synthesis of pyrazine carboxamides.

The target compounds were synthesized by microwave assisted coupling reaction of methyl ester of substituted pyrazine carboxylic acids (**2**) with ring substituted benzylamines (**3**) which yielded series of substituted* N-*benzyl pyrazine-2-carboxamides (**4a**–**j**) (Scheme 1). They used hydrophobic electron withdrawing (halogens), alkyl substituents on the pyrazine (methyl,* t-*butyl), and their combination of substituents (alkyl, alkoxy, acetyl, OH, and halogens) on benzene part.

The antimycobacterial evaluation of the compounds showed no improvement in comparison with pyrazinamide. The most active compound in this series is compound** 4c** (MIC, 25 *μ*g/mL) against pyrazinamide (MIC 6.25 *μ*g/mL).

**Scheme 2:**
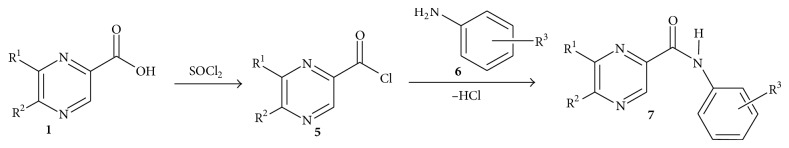
Synthesis of new derivatives of pyrazine carboxamides.

Dole$\overset{ˇ}{\text{z}}$al et al. [49] further reported the synthesis of new derivatives of* N*-phenyl pyrazine-2-carboxamide (**7a**–**l**) with improved antimycobacterial activity. They achieved this by reacting pyrazine-2-carboxylic, 6-chloropyrazine-2-carboxylic, 5-*tert-*butylpyrazine-2-carboxylic, or 5-*tert-*butyl-6-chloropyrazine-2-carboxylic acid, respectively, (50 mmol) with thionyl chloride (5.5 mL, 75.0 mmole) in dry toluene (20 mL) on reflux for 1 h. They removed the excess thionyl chloride by repeated evaporation with dry toluene in vacuo. The crude acyl chloride (**5**) dissolved in dry acetone (50 mL) was added drop wise to a stirred solution of the corresponding substituted amine (**6**) (50 mL) and pyridine (50 mmole) in dry acetone (50 mL) kept at room temperature. After the addition was completed, stirring was continued for 30 min, and then the reaction mixture was poured into cold water (100 mL) and the crude amide was collected and purified by the column chromatography (Scheme 2).

**Figure 1:**
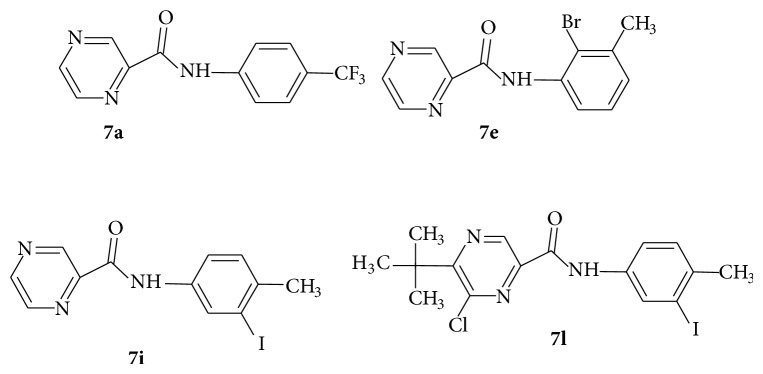
SAR of tetrahydropyrazolopyrimidine carboxamides.

The antimycobacterial activity screening of the twelve compounds showed that several novel derivatives had relatively higher activity against* M. tuberculosis, *namely,* N-*(4-trifluoromethyl phenyl) pyrazine-2-carboxamide,* N-*(2-bromo-3-methylphenyl) pyrazine-2-carboxamide, and* N-*(3-iodo-4-methylphenyl) pyrazine-2-carboxamide. These structures exhibited minimum inhibitory concentrations <2 mg/L. They also carried out antimycobacterial evaluation at the tuberculosis antimicrobial acquisition and coordinating facility (TAACF) program. 5-*tert-*Butyl-6-chloro-*N-*(3-iodo-4-methylphenyl) pyrazine-2-carboxamide was the most active compound at the TAACF antituberculosis screening (IC_90_ = 0.819 *μ*g/mL). In the SAR, the importance of iodine substitution in position 3 of benzene ring for the antimycobacterial activity was identified, mostly in compounds** 7i** and** 7l**. The discrepancy between the results of two antimycobacterial assays was explained by using different laboratory conditions (pH, growth medium). Acidic pH (pH 5.5) is crucial for the mode of action of PZA where PZA as a prodrug is converted into active form of pyrazinoic acid inside the bacilli [50]. Although compounds** 7a**,** 7e**,** 7i**, and** 7l** (Figure 1) had better antitubercular activity (MIC of 2, 2, <2 and 4 mg/L) than pyrazinamide (MIC 8 mg/L) in the experiment performed at the Czech Republic, only compound** 7l** (IC_90_ 0.819 mg/mL) maintained its lead against pyrazinamide (IC_90_ > 20^b^) [51] when the experimental conditions were changed.

## 3. Synthesis of* N-*[(2^**I**^-Substituted Phenyl)-1,3^**I**^-thiazol-5-one]-naphtho[2,1-b]furan-2-carboxamide Derivatives

**Scheme 3:**
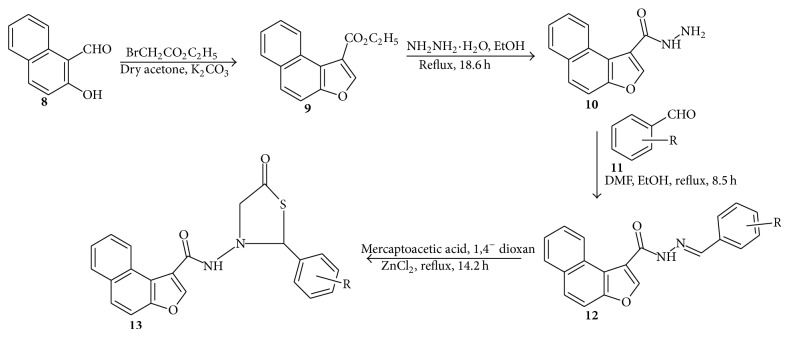
Synthesis of naphthofuran carboxamides.

Murugan et al. [52] reported the synthesis of* N-*[(2^I^-substituted phenyl)-1,3^I^-thiazol-5-one]-naphtho[2,1-b]furan-2-carboxamide derivatives (**12–15**). A mixture of 2-hydroxy-1-naphthaldehyde (**8**), ethyl bromoacetate, and anhydrous potassium carbonate was heated under reflux for 24.35 h. The reaction mixture was filtered and potassium carbonate was washed with acetone which was evaporated to get carboxylate (**9**). To this point, hydrazine hydrate and ethanol were added and refluxed for 18.3 h. The excess ethanol was distilled off to get the respective carbohydrazide (**10**). The carbohydrazide** 10 **was mixed with a solution of various substituted aromatic aldehydes (**11**) in ethanol in DMF. The reaction mixture was refluxed for 8.2 h, cooled to room temperature, and poured into crushed ice to yield carboxamide (**12**). To the carboxamide** 12** in 1,4-dioxane, mercaptoacetic acid and catalytic amount of anhydrous zinc chloride were added. The mixture was refluxed for 4.4 h, cooled, and poured into sodium bicarbonate solution to remove unreacted mercaptoacetic acid which was filtered to get the final products (**14**–**17**) (Scheme 3).

**Figure 2:**
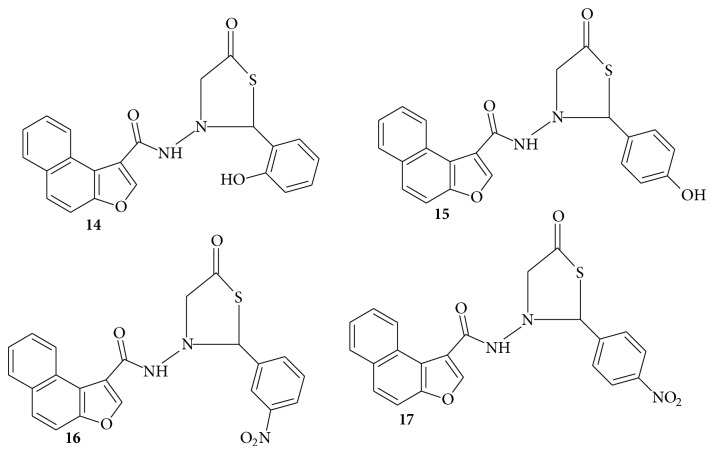


The antitubercular activities of the compounds were assessed against* M. tuberculosis* using microplate AlamarBlue assay (MABA). They reported four (**14**–**17**) (Figure 2) of the tested compounds to be active at concentrations of 50 and 100 *μ*g/mL (Table 1).

## 4. Synthesis of* N,N*-Diaryl-4-(4,5-dichloroimidazole-2-yl)-1,4-dihydro-2,6-dimethyl-3,5-pyridine Dicarboxamides

The dihydropyridines (DHPs) are well known drugs for the treatment of hypertension and cardiovascular disorders [53]. In addition, 1,4-DHP class of compounds is excellent synthon for the development of antitubercular agents [54–56]. It has been demonstrated previously that substitution of arylamide group for dicarboxylic ester moiety reduces the Ca^2+^ channel blocker activity and increases antitubercular activity [57].

**Scheme 4:**
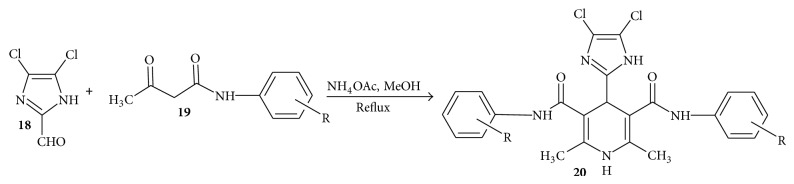
Synthesis of pyridine dicarboxamides.

In continuation of search for 1,4-DHPs with improved antitubercular activity, Gaveriya et al. [58] synthesized* N,N*-diaryl-4-(4,5-dichloroimidazole-2-yl)-1,4-dihydro-2,6-dimethyl-3,5-pyridine dicarboxamides (**20a**–**j**). The diaryls were synthesized by condensation of 4,5-dichloroimidazole-2-carboxaldehyde (**18**),* N*-aryl acetoacetamide (**19**), and ammonium acetate in methanol. 4,5-Dichloroimidazole-2-carboxaldehyde** 18** was prepared according to literature [59] and* N-*aryl acetoacetamides** 19 **according to modified Clemens method [60] by simple condensation of 2,2,6-trimethyl-1,3-dioxin-4-one with appropriate aryl amine (Scheme 4).

They tested all compounds against* M. tuberculosis *H_37_Rv strain at the concentration of 6.25 *μ*g/mL using DMSO as a solubilizing agent. The antitubercular activity result indicated that the substitution of 4,5-dichloroimidazole ring at 4-position of 1,4-DHP affects the antitubercular activity when 3,5-diester group in classic DHP structure was replaced by carboxamide moiety. On comparison, the most active compound is** 20d** with 3-chlorophenyl group at 3,5-dicarboxamide position. 3-Nitrophenyl and 4-nitrophenyl substituted compounds were also relatively active, but other substitutions did not show good activity. Although none of the new compounds had antibacterial activity comparable with rifampicin, the results serve as valuable probes to study the structure function relationship for antitubercular activity.

## 5. Synthesis of Novel Thiadiazolyl Pyrrolidine Carboxamides

A new direction in the synthesis of antitubercular agents is directed on the design of molecules acting as enzyme inhibitors. The target enzyme should play a vital role in any phase of the life cycle of the pathogen and should be absent in the host. Enoyl-acyl carrier protein reductase is a FAS II enzyme involved in the bacterial fatty acid biosynthetic pathway in the mycobacterium and other bacteria [61]. These enzymes are involved in fatty acid elongation in the cell wall synthesis. The prime TB drug isoniazid is reported to be a potent enoyl-ACP reductase inhibitor but requires initial activation by* Kat G*, a catalase peroxidase enzyme [62]. This activation step necessitated the search for new antitubercular agents which can act as direct enoyl ACP reductase inhibitors. This prompted Boyne et al. [63] to synthesize thiadiazolyl pyrrolidine carboxamides (**26a**–**e**) and tested their enoyl ACP reductase inhibition activity.

**Scheme 5:**
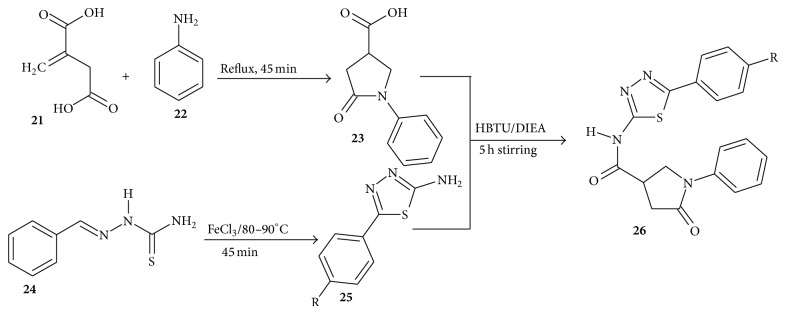
Synthesis of thiazolyl pyrrolidine carboxamides.

In their synthesis, 5-oxo-1-phenylpyrrolidine-3-carboxylic acid** 23** was synthesized by refluxing a mixture of itaconic acid [**21**], aniline [**22**], and water for 1 h or until the odour of aniline becomes faint after which the reaction was chilled for 1 h. The synthesis of 2-amino-5-(4-substituted)phenyl aryl-1,3,4-thiadiazole** 25** was achieved by dissolving aromatic aldehyde and thiosemicarbazide, respectively, in warm alcohol and warm water and mixing the two solutions slowly with stirring. The target compounds were synthesized by dissolving compounds** 23 **and** 25 **in dry DMF. HBTU and DIEA were added and the mixture was stirred for 5 h at 23°C. The reaction was quenched using NaCl solution and the mixture extracted with ethyl acetate. The combined ethyl acetate layer was washed with 1N HCl and then with saturated sodium bicarbonate followed by brine (Scheme 5).

The antimycobacterial activities of the compounds were assessed against* M. tuberculosis* using MABA. The antitubercular activities are as presented in Table 2.

## 6. Synthesis of Substituted* N*-Phenyl-6-methyl-2-oxo-4-phenyl-1,2,3,4-tetrahydro Pyrimidine-5-carboxamides

Within the pyrimidines, 2,4-diaminopyrimidines have been reported to have IC_50_ of 0.0058 *μ*M and a safety index >600 [64]. The most effective derivative in the chloropyrimidine series has an MIC of 0.78 *μ*g/mL [65], while the most successful compound from the anilinopyrimidine series displayed an MIC of 3.12 *μ*g/mL [66] Thymidine monophosphate derivatives have been evaluated for binding to thymidine monophosphate kinase of* M. tuberculosis.* The most effective inhibitor of this class has a Ki of 10.5 *μ*M [67]. These results prompted Vanheusden et al. [68] to synthesize series of* N*-phenyl-6-methyl-2-oxo-4-phenyl-1,2,3,4-tetrahydropyrimidine-5-carboxamides [**31a**–**v**,** 32a**–**g**] and evaluate their antimycobacterial activity.

**Scheme 6:**
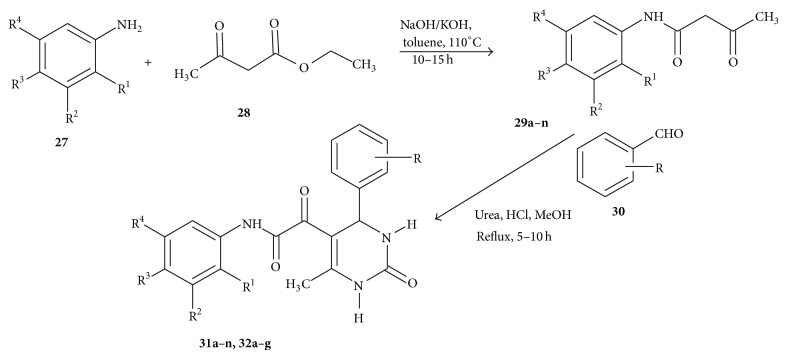
Synthesis of pyrimidine carboxamides.

Virsodia et al. [69] carried out the synthesis of the target compounds utilizing various substituted acetoacetanilides (**29a**–**n**). Compounds (**29a**–**n**) were synthesized by reacting substituted amines and ethyl acetoacetate in toluene with a catalytic amount of NaOH or KOH (Scheme 6). The reaction mixture was heated at 120°C for 10–15 h. Fourteen different acetoacetanilides were synthesized bearing various electron withdrawing and electron donating groups like 2,3-diCH_3_, 3,4-diCH_3_, 4-CH_3_, H, 2,5-diCH_3_; 2,4-diCH_3_; 3-Cl-4-F; 4-F; 4-Cl; 2-F; 4-OCH_3_, 2,5-diCl, and 3-NO_2_ on the phenyl ring. Acetoacetanilides, thus obtained, were used as 1,3-diketone adducts for the multicomponent Biginelli reaction.

The acetoacetanilides (**29a**–**n**) were reacted with substituted aldehydes and urea in methanol using concentrated HCl in catalytic amount to obtain the title compounds (**31a**–**v**,** 32a**–**g**) as depicted in Scheme 6.

The antitubercular activities of the compounds were tested against* M. tuberculosis* H_37_Rv strain. Percentage inhibition data of compounds (**31a**–**v**,** 32a**–**g**) are reported in Table 3. Compounds** 31c** and** 32f**, with dimethyl phenyl and 3,4-dimethylcarbamoyl side chain, respectively, showed 65% and 63% inhibition. Thus, methyl group at these positions showed higher potency. But substitutions on 4-phenyl ring also alter the activity of compound. Compound** 31m **was having 3,4-dimethylphenyl carbamoyl side chain as in compound** 32f**, but NO_2_ group is at* meta*-position in compound** 31m** which leads to a decrease in % inhibition from 63% to 13%. Thus, compounds with methyl substitution on phenyl carbamoyl side chain with –OPh or –NO_2_ substitution at* meta-*position of 4-phenyl ring were more potent than the same substitution on* para*-position. The replacement of methyl group in phenyl ring of phenyl carbamoyl side chain with halogens results in the loss of antitubercular activity. Compounds with halogen substituted at different positions of phenyl ring of phenyl carbamoyl side chain do not show good potency either with* meta-* or with* para*-substituted 4-phenyl ring of C_5_ side chain with* meta*-substituted 4-phenyl ring showing good potency.

## 7. Synthesis of Aryl Thiazolidine Carboxamides

Sriram et al. [70] synthesized 2-(substituted aryl)-*N-*(substituted) thiazolidine-4-carboxamides** 35(a**–**d)**–**50(a**–**d)**. The compounds were synthesized from 2-(substituted aryl)-*N-*(substituted) thiazolidine-4-carboxamides (**34a**–**d**). 2-(Substituted aryl)-*N-*(substituted) thiazolidine-4-carboxylic acids were synthesized as follows. Potassium acetate was added to a solution of L-cysteine hydrochloride** 33** in water. To this homogenous mixture, ethanol and appropriate aldehyde** 30** were added. The reaction was stirred below 25°C for 6 h. The solid that precipitated was filtered and washed with cold ethanol and dried to afford** 34a**–**d**.

**Scheme 7:**
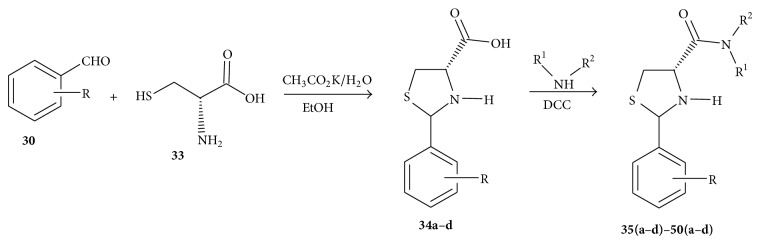
Synthesis of aryl thiazolidine carboxamides.

They synthesized the carboxamides** 35(a**–**d)**–**50(a**–**d**), by mixing appropriate carboxylic acid** 34a**–**d** and DCC in dichloromethane, and stirred them for 10 min at 0°C. To this mixture, appropriate primary or secondary amine was added and stirred for 8 h. The solid urea separated was filtered off and the organic layer was washed with water and dried over sodium sulphate and distilled under reduced pressure to yield the desired product (Scheme 7).

**Figure 3:**
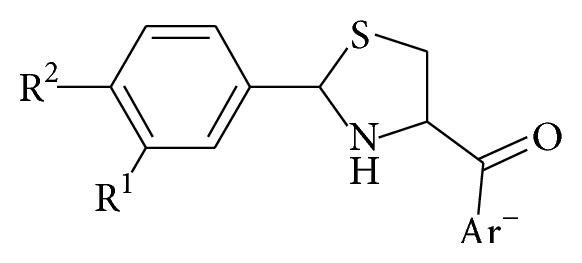


The compounds were screened for their* in vitro* antimycobacterial activity against* M. tuberculosis* (MTB) and* M. smegmatis *ATCC 14468 (MC2) by agar dilution method for the determination of MIC in duplicate. The result of the MIC is as given in Table 4. The structural core is presented in Figure 3.

As could be read from Table 4, all the compounds prepared showed excellent* in vitro* activity against MTB with MICs ranging from 0.12 to 20.94 *μ*M. Seventeen compounds (**39a**,** 46a**,** 47a**,** 50a**,** 37b**,** 40b**,** 41b**,** 43b**,** 45b**,** 49b**,** 50b**,** 36d**,** 39d**,** 45d**,** 46d**,** 47d**,and** 50d**) had MIC less than 1 *μ*M. When compared to isoniazid (MIC: 0.66 *μ*M), thirteen compounds (**39a**,** 37b**,** 40b**,** 41b**,** 43b**,** 45b**,** 49b**,** 50b**,** 36d**,** 39d**,** 46d**,** 47d**,and** 50d**) were found to be more active against MTB. Three compounds (**43b**,** 47d**, and** 50d**) were found to be more potent than rifampicin (MIC: 0.23 *μ*M). Compound** 43b** was found to be the most active compound* in vitro* with MIC of 0.12 *μ*M against MTB and it was 5.5 and 1.9 times more potent than isoniazid and rifampicin, respectively.

With respect to structural antitubercular activity, in the carboxamide end, they prepared various phenyl (**35**–**39**), pyridyl (**41-42**), arylpiperazine (**43**–**45**), and fluoroquinolone (**46**–**50**) side chain. Among them, the order of activity from Table 4 is fluoroquinolone > arylpiperazine > pyridyl > phenyl side chain. Among the phenyl ring, dinitro substituents showed excellent activity and the order of activity is 2,4-(NO_2_)_2_ > 4-Cl > 4-CH_3_ > 4-CF_3_ > 6-CH_3_ > H. In the case of aryl ring, halogen showed good activity and the order of activity is as follows: 4-Cl > 5-CH_3_> 4-CH_3_. In the case of aryl ring of piperazine derivatives, one can see benzyl > 4-chlorophenyl > phenyl. Among the fluoroquinolones, the order of activity is moxifloxacin > gatifloxacin > ciprofloxacin > norfloxacin > lomefloxacin.

## 8. Synthesis of Phenothiazine Derived Thiazolidinone Carboxamides

Phenothiazine is a bioactive heterocyclic compound of pharmaceutical importance and possesses different biological activities, namely, antibacterial [71, 72], antifungal [73], antitubercular [74], and anti-inflammatory activities [75].

**Scheme 8:**
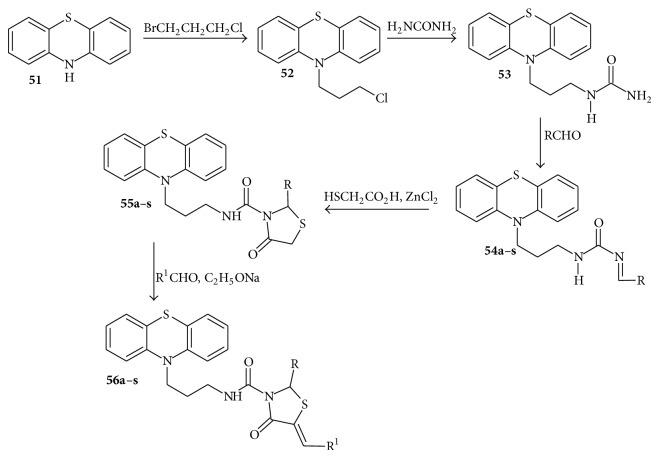
Synthesis of phenothiazine derived thiazolidinone carboxamides.

The synthesis was achieved as reported by Sharma et al. [76] as follows: the starting material, phenothiazine** 51** with 1-bromo-3-chloropropane underwent a nucleophilic substitution reaction yielding 10-(3-chloropropyl)-10*H-*phenothiazine compound** 52**. Compound** 52 **on reaction with urea afforded* N*-[3-(10*H*-phenothiazine-10-yl)propyl]urea, compound** 53**. Compound** 53 **on reaction with several selected substituted benzaldehydes underwent a condensation reaction to afford* N*-[3-(10*H*-phenothiazine-10-yl)propyl]-*N* ^1^-[(substituted phenyl)-methylidene]urea, compounds** 54a**–**s**. The reaction of thioglycolic acid with compounds** 54a**–**s **in the presence of anhydrous ZnCl_2_ gave new heterocyclic compounds* N*-[3-(10*H*-phenothiazine-10-yl)propyl]-2-(substituted phenyl)-4-oxo-3-thiazolidine carboxamide, compounds** 55a**–**s**. Compounds** 55a**–**s **on treatment with various selected substituted benzaldehydes in the presence of C_2_H_5_ONa underwent a Knoevenagel condensation reaction to yield the final products* N*-[3-(10*H*-phenothiazine-10-yl)propyl]-2-(substituted phenyl)-4-oxo-5(substituted benzylidene)-3-thiazolidine-carboxamide, compounds** 56a**–**s **(Scheme 8).

The results of the antitubercular activities are summarized in Table 5. All the compounds** 51**,** 52**,** 53a**–**s**,** 54a**–**s**, and** 55a**–**s** were screened for their antitubercular activity against* M*.* tuberculosis *(H37Rv strain). The investigation of antimicrobial data revealed that compounds** 56c**,** 56d**,** 56e**,** 56f**,** 56h**,** 56i**, and** 56j **displayed high activity, compounds** 55h**,** 55j**,** 56b**,** 56g**,and** 56q **showed moderate activity, and the other compounds showed less activity compared with standard drugs.

The compounds exhibited a structure activity relationship (SAR) because the activity of compounds varies with substitution. The nitrogroup-containing compounds** 56h**,** 56i**,and** 56j **showed higher activity than the chloro-group-(**56c **and** 56d**) or the bromo-group-containing compounds (**56e **and** 56f**). In addition, the chloro- and bromo-derivatives also had a higher activity than the other tested compounds. Based on the SAR, it could be concluded that the activity of compounds depended on the electron withdrawing nature of the substituent groups. The sequence of the activity is the following: NO_2_ > Cl > Br > OCH_3_ < OH > CH_3_.

## 9. Synthesis of Tetrahydropyrazolopyrimidine Carboxamides

**Figure 4:**
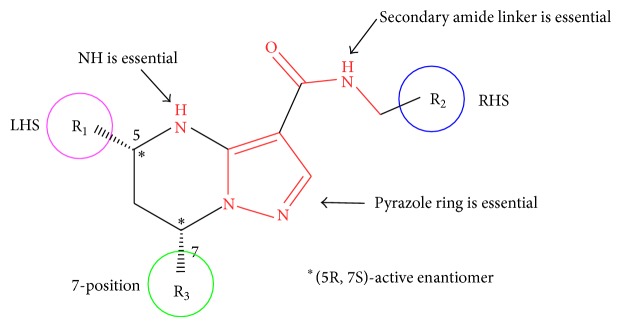


To identify a new starting point in the development of new TB drugs, the Novartis internal small molecule chemical library was screened for activity against* Mycobacterium bovis* BCG as a surrogate of* M. tuberculosis* by measuring ATP levels using the BacTiter-Glo assay as described in literature [77]. Subsequent hit confirmation with* M. tuberculosis* H37Rv led to the identification of tetrahydropyrazolo[1,5-a]pyrimidine scaffold as one of the hit series. Two other groups have also independently reported this scaffold as a hit from their own phenotypic high-throughput screening campaigns against TB [78–80]. Yokokawa et al. [81] described the synthesis of tetrahydropyrimidine carboxamides exploring the structure activity relationship (SAR) and structure-property relationship (SPR) of this class and the results of* in vivo* pharmacokinetics and pharmacological evaluation of selected compounds in mice. Their initial SAR study identified the key pharmacophore required for anti-TB activity as summarized in Figure 4. The NH of the tetrahydropyrimidine ring, the secondary amide linker, and the pyrazole ring were all found to be essential to retain low micromolar values of MIC (minimal inhibitory concentration, defined as the concentration that prevents 50% of bacterial growth at 5 days postinhibitor exposure). Significant differential anti-TB activity of the stereoisomers at the C-5 and C-7 positions was observed, and the absolute stereochemistry of the active enantiomer was confirmed to be 5R, 7S with X-ray crystal analysis. The corresponding (5S,7R) isomers were proved to be inactive (MIC > 20 *μ*M). This result indicates that the (5R,7S) form of this scaffold may interact appropriately with some pocket of the yet unknown biological target inside the TB bacteria. Next, they focused on the exploration of SAR and SPR for the phenyl left-hand side (LHS), benzyl right-hand side (RHS), and trifluoromethyl at the C-7 position to optimize the balance of potency and physicochemical properties.

**Scheme 9:**
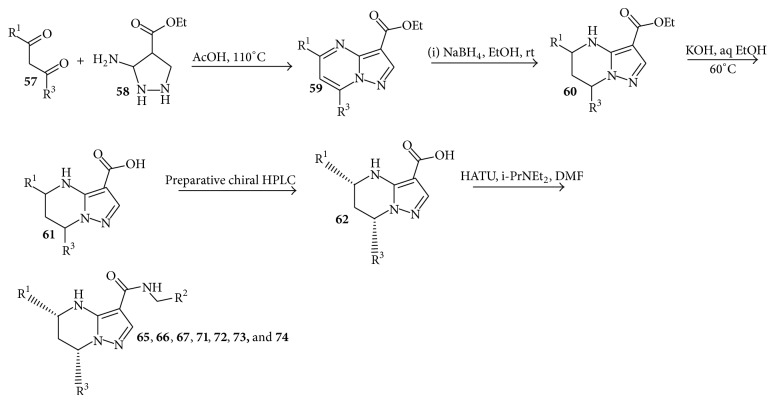
Synthesis of tetrahydropyrazolopyrimidine carboxamides.

**Scheme 10:**
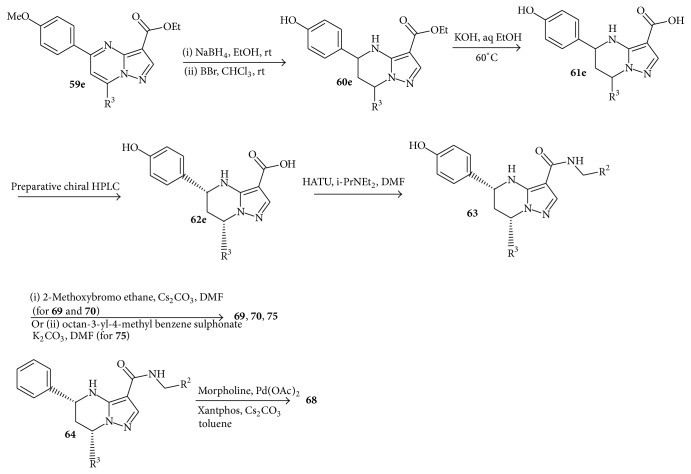
Synthesis of tetrahydropyrazolopyrimidine carboxamides.

Condensation of the aminopyrazole** 58** with the corresponding diketones** 57** in acetic acid yielded the pyrazolopyrimidines** 59** as single regioisomer at the 5,7-position. Reduction of the pyrimidine ring with sodium borohydride (NaBH_4_) afforded only the 5,7-cis isomer of the tetrahydropyrimidine analogue** 60**. The* para*-methoxy group of** 59e** was cleaved by boron tribromide (BBr_3_) to give the phenol** 60e**. Subsequently, alkaline hydrolysis of the ester** 60** with potassium hydroxide afforded the racemic acid** 61**, which was separated by preparative high performance liquid chromatography (HPLC) using a chiral column to provide the desired (5R,7S) form** 62**. Coupled with the corresponding benzyl amines using 2-(1H-7-azabenzotriazole-1-yl)-1,1,3,3-tetramethyl uranium, hexafluorophosphate (HATU) as a coupling reagent produced the target compounds** 65**,** 66**,** 67**,** 71**,** 72**,** 73**, and** 74 **as shown in Scheme 9. The synthesis of compounds** 68**,** 69**,** 70**, and** 75** is described in Scheme 10. Introduction of morpholine at the* para*-position of LHS phenyl was achieved by palladium catalyzed amination of* para*-bromophenyl of LHS 64 to afford compound** 68**. Compounds** 69**,** 70**, and** 75** were prepared by alkylation of the* para*-phenol of LHS 7 with the appropriate alkylating agents.

Compound** 65** exhibited the best potency against MTB H37Rv in the whole cell assay (MIC 0.15 *μ*M); however, it is highly lipophilic (log*P* = 6.3) and shows high plasma protein binding (>99.0%) and low aqueous solubility (<4 *μ*M at pH 6.8), which are in general unfavorable drug-like properties. To reduce the lipophilicity of the scaffold, replacement of the LHS phenyl with 2-pyridyl and 2-furyl groups led to compounds** 66** and** 67**, which were tolerated and reduced log*P* significantly (by 0.8−1.9). Introduction of polar substituents at the* para*-position of the LHS phenyl afforded compounds** 68**,** 69**, and** 70**, which also reduced log*P* and achieved anti-TB activity comparable with compound** 65 **(Table 6). Introduction of the 2- and 3-pyridyl rings on the RHS reduced log*P* without affecting the potency (compounds** 71** and** 72**). However, all of these modifications had little effect on solubility and plasma protein binding. Replacement of the core 7-trifluoromethyl substituent with difluoromethyl in** 66** afforded compound** 73**, which interestingly also increased aqueous solubility. The combination of the LHS pyridyl with RHS pyridyl generated compound** 74**, which led to a significant decrease in log*P* (3.3) and thereby increased intrinsic aqueous solubility (0.21 g/L). However, this modification also resulted in the loss of anti-TB activity (MIC = 52.2 *μ*M). Compound** 75** suffered from modest anti-TB activity despite its improved physicochemical properties. Compound** 65** showed a potent bactericidal effect and activity in an* in vitro* macrophage model. Furthermore,** 65** is active across all MDR-TB isolates suggesting a novel mechanism of action. Studies to elucidate a mechanism of action of this series will be discussed elsewhere [82]. The* in vivo* pharmacokinetics (PK) of compounds** 65**,** 66**,** 70**, and** 73 **were evaluated in mice by oral (po) and intravenous (iv) routes at doses of 25 and 5 mg/kg, respectively. All four compounds displayed low to moderate total systemic clearance and volume of distribution with elimination half-lives ranging from 1.3 to 4 h. These compounds showed good oral bioavailability (45−100%) and good oral exposure in systemic circulation. In addition, these compounds exhibited no significant CYP inhibition (based on reversible inhibition assays using midazolam for CYP 3A4/5, bufuralol for CYP2D6, and diclofenac for CYP 2C9 as markers) and induction.

## 10. Conclusion

This work has reviewed the synthesis and antitubercular activities of over two hundred carboxamide derivatives. In most of the synthesis reported, there was almost always a comparison between the antitubercular activities of the novel compounds with isoniazid, rifampicin, or pyrazinamide. The review reveals the following compounds as being more active than isoniazid, rifampicin, or pyrazinamide. From the work of Dole$\overset{ˇ}{\text{z}}$al et al. [49], it was shown that, from the antitubercular activity carried out at Czech Republic, compounds** 7a**,** 7e**,** 7i**, and** 7l** were more potent against* M. tuberculosis *than pyrazinamide but only** 7l** was found to be more active than pyrazinamide when the IC_90_ was carried out at TAACF USA. The work of Sriram et al. [70] also revealed that compounds** 39a**,** 37b**,** 40b**,** 41b**,** 43b**,** 45b**,** 49b**,** 50b**,** 36d**,** 39d**,** 46d**,** 47d**, and** 50d **were more active than isoniazid whereas compounds** 43b**,** 47d**, and** 50d** were found to be more active than rifampicin. Since rifampicin (MIC 0.23 *μ*M) is more active than isoniazid (MIC 0.66 *μ*M), it can be said categorically that only three of the one hundred and thirty-four new derivatives of carboxamide reviewed were found to be more active than the existing antitubercular agents. The most active compound is** 43b** (MIC 0.12 *μ*M). Yokokawa et al. [81] also revealed compound** 65 **withMIC 0.15 *μ*M and** 67 **with 0.13 *μ*M.

## Figures and Tables

**Table 1:** MIC of naphthofuran carboxamides.

Compd. number/conc. µg/mL	100	50	25	12.5	6.25	3.125	1.6	0.8	0.2
**14**	S	S	R	R	R	R	R	R	R
**15**	S	S	R	R	R	R	R	R	R
**16**	S	S	R	R	R	R	R	R	R
**17**	S	S	R	R	R	R	R	R	R

S = sensitive, R = resistance.

**Table 2:** SAR and MIC of thiazolyl pyrrolidine carboxamides.

Compd. number	MIC (µg/mL)	R
**26a**	25	H
**26b**	50	Cl
**26c**	50	CH_3_
**26d**	25	OCH_3_
**26e**	25	NO_2_

**Table 3:** SAR and MIC of pyrimidine carboxamides.

Compd. number	R	R^1^	R^2^	R^3^	R^4^	% inhibition (µg/mL)
**31a**	4-OCH_3_	CH_3_	H	H	CH_3_	2
**31b**	3-OPh	CH_3_	H	H	CH_3_	27
**31c**	3-OPh	CH_3_	CH_3_	H	H	65
**31d**	2-NO_2_	Cl	H	H	H	11
**31e**	4-NO_2_	CH_3_	H	H	CH_3_	4
**31f**	4-Cl	H	H	H	H	6
**31g**	4-OH	F	H	H	H	18
**31h**	4-NO_2_	Cl	H	H	Cl	18
**31i**	4-OH	CH_3_	H	CH_3_	H	12
**31j**	4-OH	H	NO_2_	H	H	2
**31k**	3-Cl	H	Cl	F	H	48
**31l**	4-NO_2_	F	H	H	H	4
**31m**	4-NO_2_	H	CH_3_	CH_3_	H	13
**31n**	4-NO_2_	CH_3_	H	CH_3_	H	12
**31o**	3-NO_2_	Cl	H	H	H	26
**31p**	3-NO_2_	H	Cl	F	H	29
**31q**	3-NO_2_	F	H	H	H	24
**31r**	3-Cl	H	H	F	H	38
**31s**	4-NO_2_	H	H	OCH_3_	H	21
**31t**	3-NO_2_	H	H	Cl	H	29
**31u**	3-NO_2_	H	H	CH_3_	H	28
**31v**	3-NO_2_	H	H	F	H	30

**32a**	3-NO_2_	CH_3_	H	H	H	6
**32b**	4-Cl	Cl	H	H	H	26
**32c**	4-NO_2_	H	H	Cl	H	9
**32d**	3-OPh	H	CH_3_	CH_3_	H	32
**32e**	4-NO_2_	H	H	H	H	25
**32f**	3-NO_2_	H	CH_3_	CH_3_	H	63
**32g**	4-NO_2_	H	Cl	F	H	22

**Table 4:** SAR and MIC of aryl thiazolidine carboxamides.

Number	Ar	R^1^	R^2^	MTB	MC2
**35a**	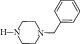	H	H	11.00	11.00
**35b**	-do-	H	F	5.15	5.19
**35c**	-do-	H	NO_2_	20.94	20.94
**35d**	-do-	OCH_3_	OH	18.91	9.47
**36a**	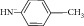	H	H	1.34	2.64
**36b**	-do-	H	F	4.93	39.50
**36c**	-do-	H	NO_2_	10.01	5.02
**36d**	-do-	OCH_3_	OH	0.58	2.29
**37a**	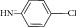	H	H	9.81	19.60
**37b**	-do-	H	F	0.59	2.34
**37c**	-do-	H	NO_2_	8.60	17.17
**37d**	-do-	OCH_3_	OH	1.09	4.30
**38a**	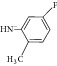	H	H	4.93	19.75
**38b**	-do-	H	F	4.66	9.36
**38c**	-do-	H	NO_2_	9.47	18.91
**38d**	-do-	OCH_3_	OH	2.15	8.63
**39a**	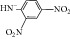	H	H	0.53	2.08
**39b**	-do-	H	F	1.01	7.97
**39c**	-do-	H	NO_2_	7.46	29.80
**39d**	-do-	OCH_3_	OH	0.47	1.87
**40a**	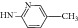	H	H	20.87	20.87
**40b**	-do-	H	H	0.63	2.48
**40c**	-do-	H	NO_2_	9.08	18.14
**40d**	-do-	OCH_3_	OH	1.15	2.28
**41a**	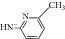	H	H	20.87	10.45
**41b**	-do-	H	F	0.63	2.48
**41c**	-do-	H	NO_2_	4.52	18.14
**41d**	-do-	OCH_3_	OH	4.51	4.51
**42a**	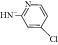	H	H	9.78	4.87
**42b**	-do-	H	F	1.18	4.64
**42c**	-do-	H	NO_2_	4.27	17.13
**42d**	-do-	OCH_3_	OH	4.26	4.26
**43a**	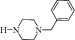	H	H	4.24	2.14
**43b**	-do-	H	F	0.12	2.04
**43c**	-do-	H	NO_2_	3.78	15.15
**43d**	-do-	OCH_3_	OH	1.88	1.88
**44a**	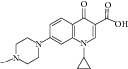	H	H	17.68	35.36
**44b**	-do-	H	F	1.07	2.12
**44c**	-do-	H	NO_2_	7.85	15.68
**44d**	-do-	OCH_3_	OH	7.83	18.09
**45a**	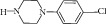	H	H	16.11	8.06
**45b**	-do-	H	F	0.49	1.94
**45c**	-do-	H	NO_2_	1.80	3.62
**45d**	-do-	OCH_3_	OH	0.92	1.82
**46a**	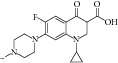	H	H	0.76	1.51
**46b**	-do-	H	F	1.44	5.79
**46c**	-do-	H	NO_2_	5.51	11.01
**46d**	-do-	OCH_3_	OH	0.35	1.38
**47a**	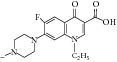	H	H	0.78	1.54
**47b**	-do-	H	F	2.95	2.97
**47c**	-do-	H	NO_2_	5.63	5.63
**47d**	-do-	OCH_3_	OH	0.17	1.41
**48a**	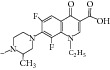	H	H	2.88	11.56
**48b**	-do-	H	F	2.79	5.60
**48c**	-do-	H	NO_2_	5.34	10.67
**48d**	-do-	OCH_3_	OH	1.32	5.33
**49a**	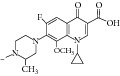	H	H	2.75	11.02
**49b**	-do-	H	F	0.34	1.35
**49c**	-do-	H	NO_2_	2.55	5.11
**49d**	-do-	OCH_3_	OH	1.27	5.10
**50a**	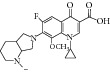	H	H	0.67	1.23
**50b**	-do-	H	F	0.32	1.23
**50c**	-do-	H	NO_2_	2.44	1.23
**50d**	-do-	OCH_3_	OH	0.15	2.45
INH				0.66	>123
RIFAM PICIN				0.23	45.57
Ciprofloxacin				4.71	2.35

**Table 5:** SAR and MIC of phenothiazine derived thiazolidinone carboxamides.

Compd. number	50 µg/L inhibition (%)	25 µg/L inhibition (%)	R = R^1^
**52**	20	13	
**53**	18	10	
**54a**	22	18	C_6_H_5_
**54b**	32	25	4-ClC_6_H_4_
**54c**	34	27	3-ClC_6_H_4_
**54d**	35	30	2-ClC_6_H_4_
**54e**	40	28	4-BrC_6_H_4_
**54f**	50	27	3-BrC_6_H_4_
**54g**	52	25	2-BrC_6_H_4_
**54h**	65	32	4-NO_2_C_6_H_4_
**54i**	68	35	3-NO_2_C_6_H_4_
**54j**	66	38	2-NO_2_C_6_H_4_
**54k**	40	25	4-CH_3_OC_6_H_4_
**54l**	42	28	3-CH_3_OC_6_H_4_
**54m**	43	23	2-CH_3_OC_6_H_4_
**54n**	38	20	4-CH_3_C_6_H_4_
**54o**	35	24	3-CH_3_C_6_H_4_
**54p**	38	25	2-CH_3_C_6_H_4_
**54q**	50	28	4-HOCH_3_
**54r**	52	30	3-HOCH_3_
**54s**	55	32	2-HOCH_3_
**55a**	35	20	C_6_H_5_
**55b**	55	25	4-ClC_6_H_4_
**55c**	60	30	3-ClC_6_H_4_
**55d**	60	30	2-ClC_6_H_4_
**55e**	68	30	4-BrC_6_H_4_
**55f**	70	32	3-BrC_6_H_4_
**55g**	75	30	2-BrC_6_H_4_
**55h**	70	30	4-NO_2_C_6_H_4_
**55i**	68	35	3-NO_2_C_6_H_4_
**55j**	70	35	2-NO_2_C_6_H_4_
**55k**	50	30	4-CH_3_OC_6_H_4_
**55l**	53	32	3-CH_3_OC_6_H_4_
**55m**	50	30	2-CH_3_OC_6_H_4_
**55n**	41	29	4-CH_3_C_6_H_4_
**55o**	42	28	3-CH_3_C_6_H_4_
**55p**	45	30	2-CH_3_C_6_H_4_
**55q**	70	33	4-HOCH_3_
**55r**	70	34	3-HOCH_3_
**55s**	65	33	2-HOCH_3_
**56a**	45	22	C_6_H_5_
**56b**	74	32	4-ClC_6_H_4_
**56c**	80	36	3-ClC_6_H_4_
**56d**	80	32	2-ClC_6_H_4_
**56e**	78	30	4-BrC_6_H_4_
**56f**	79	30	3-BrC_6_H_4_
**56g**	76	29	2-BrC_6_H_4_
**56h**	82	32	4-NO_2_C_6_H_4_
**56i**	83	27	3-NO_2_C_6_H_4_
**56j**	81	28	2-NO_2_C_6_H_4_
**56k**	60	28	4-CH_3_OC_6_H_4_
**56l**	63	30	3-CH_3_OC_6_H_4_
**56m**	65	31	2-CH_3_OC_6_H_4_
**56n**	45	22	4-CH_3_C_6_H_4_
**56o**	49	18	3-CH_3_C_6_H_4_
**56p**	47	20	2-CH_3_C_6_H_4_
**56q**	76	24	4-HOCH_3_
**56r**	70	27	3-HOCH_3_
**56s**	65	25	2-HOCH_3_
Rifampicin		100	
Isoniazid		100	

**Table 6:** SAR and MIC of tetrahydropyrazolopyrimidine carboxamides.

Compounds	R^1^	R^2^	R^3^	MIC (µM)	log⁡*P* ^a^	Solubility^b^ (mM, pH 6.8)	PPB (%)^c^(h/m)
**65**	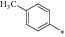	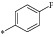	CF_3_	0.15 ± 0.04	6.3	<4	>99.0/99.0

**66**	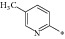	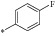	CF_3_	0.44 ± 0.1	4.4	9	96.1/95.8

**67**	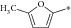	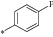	CF_3_	0.13 ± 0.06	5.5	<4	>99.0/99.0

**68**	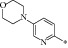	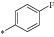	CF_3_	0.87 ± 0.18	4.8	<4	98.2/98.4

**69**	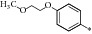	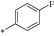	CF_3_	0.44 ± 0.04	4.1	6	98.1/98.5

**70**	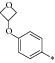	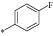	CF_3_	0.73 ± 0.1	5.1	9	97.3/97.6

**71**	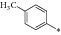	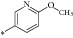	CF_3_	0.47 ± 0.15	5.1	<4	>99.0/99.0

**72**	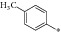	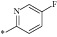	CF_3_	0.83 ± 0.24	4.9	<4	>99.0/99.0

**73**	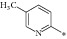	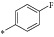	CHF_2_	0.60 ± 0.2	4.6	212	95.7/94.8

**74**	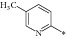	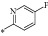	CF_3_	52.2 ± 24.1	3.2	347	81.9/87.2

**75**	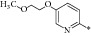	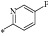	CF_3_	3.7 ± 0.6	3.2	20	87.8/89.2

^a^log⁡*P*: high throughput measured octanol/water partition coefficient, ^b^solubility: high throughput equilibrium solubility, ^c^PPB: plasma protein binding measured by rapid equilibrium dialysis (RED) device.
